# Influence of Pressure Extraction Systems on the Performance, Quality and Composition of Virgin Almond Oil and Defatted Flours

**DOI:** 10.3390/foods10051049

**Published:** 2021-05-11

**Authors:** José M. Roncero, Manuel Álvarez-Ortí, Arturo Pardo-Giménez, Adrián Rabadán, José E. Pardo

**Affiliations:** 1 Higher Technical School of Agricultural and Forestry Engineering, University of Castilla-La Mancha, Campus Universitario, s/n, 02071 Albacete, Spain; manuel.alvarez@uclm.es (M.Á.-O.); adrian.rabadan@uclm.es (A.R.); jose.pgonzalez@uclm.es (J.E.P.); 2 Mushroom Research, Experimentation and Service Centre, C/Peñicas, s/n, Apartado 63, 16220 Quintanar del Rey, Spain; apardo.cies@dipucuenca.es

**Keywords:** virgin almond oil, chemical composition, quality parameters, consumer preference, fatty acids, sterols, polyphenols

## Abstract

Almond is the most cultivated nut throughout the world. The oil content of almonds in most varieties exceeds 50%, which encourages the oil extraction to be used in gastronomy or in the cosmetic industry. The preferred system to extract almond oil is by means of pressure, which leads to obtaining a virgin oil ready for consumption. In this work, almond oil has been obtained using two pressure systems: screw press (SP) and hydraulic press (HP). The performance of both methods, as well as their influence on quality and composition characteristics of the almond oils obtained are analyzed from both a physical-chemical and sensory point of view. From an industry perspective, the highest oil yield is obtained with the SP when it operates at temperatures of 100–150 °C. Regarding the quality and chemical composition, the oils obtained by HP showed better quality indices, as they are subjected to a less aggressive treatment without influence of temperature, but lower content in total sterols. Fatty acid pattern, characterized by the predominance of unsaturated fatty acids (>90%), was not affected by the pressing system. The different operational conditions tested did not greatly affect the performance or composition of the oils obtained, but sensory tests showed two clearly differentiated products, the oil obtained by HP and that obtained by SP, according to consumer preferences. The defatted almond flours obtained as a by-product of the oil extraction process are characterized by a high content in protein and fiber, and a higher content in fat when the flour is produced from the pressing cake of HP.

## 1. Introduction

The almond is the most important nut crop in the world in terms of its commercial production, which exceeds 3 million tons annually [1]. Almond kernel is a nutrient dense food, with a high content in proteins (12–22%) or carbohydrates (20%) [2,3], rich in fiber and minerals such as magnesium and copper [4]. In addition, similar to many other nuts, the almond stands out for its high oil content (40–67%), which is characterized by a fatty acid profile with a low percentage of saturated fats, and where monounsaturated fatty acids predominate [5]. Almond oil also contains minor phytochemical compounds such as tocopherols, sterols, or phenolic compounds with biological activity and antioxidant properties [6].

Almond consumption has been associated with many health benefits [7], especially related to the reduction of cardiovascular disease risk, but also with effects on other pathologies, such as hypertension, diabetes mellitus, or metabolic syndrome. These activities are generally attributed to the lipid fraction, where the fatty acid profile has a predominant role, but also minor compounds such as polyphenols and phytosterols may be involved.

Edible oils play an important role in the food industry due to their nutritional properties and their influence on the smell and taste of food. The oils obtained from nuts are a culinary ingredient that provides new organoleptic properties [8]. In recent years, the production of high-quality almond oil for human consumption has been developed, basically marketed in Gourmet-type establishments for use in gastronomy, to accompany salads, white meats or pasta [9]. Although not described by the current Codex Alimentarius Fats and Oils Committee, almond oil is produced on a small scale in many countries such as France, Spain, and the United States. The increase in world almond production and the demand for new oil specialties encourages the investigation of appropriate methods to improve the production of edible almond oil. Its quantitative and qualitative extraction is essential to determine the feasibility of converting it into a commercial production [10].

To obtain it, solvent extraction, extraction with supercritical fluids (CO_2_), and pressure systems (hydraulic and screw presses) are usually used. The highest industrial performance, but also the worst quality of the oils, is achieved through the use of solvents [11]. In this case, oils must be refined to eliminate the rests of solvents, so they lose the condition of “virgin.” Extraction with supercritical fluids gives rise to higher quality oils [12], but at a very high price. For this purpose, physical methods are preferred, especially, cold press methods. Press extraction becomes the best extraction option, as high-quality oils are obtained at an affordable price [13,14,15]. Normally, a screw press (SP) is used as a small-volume production, and a hydraulic press (HP) is the main method for mass production. However, the optimization of this process including the evaluation of the chemical and organoleptic characteristics of the oils obtained under different conditions is hardly covered in scientific papers. Regarding the screw press, although it is generally defined as a cold-pressing system, it requires a temperature increase (preheating) to obtain better results. This increase produces a better oil separation, which can affect extraction yield [16].

Once the oil extraction is produced, a pressing cake remains with the non-lipid fraction of the almond. These by-product is generally rich in proteins, minerals, dietary fiber, and substances with antioxidant capacity [17], so it may be used to promote a healthy diet. Moreover, recent studies have explored the effect of some of these nutritional compounds like fiber on gut microbiota [18] or the antioxidant capacity of the protein fraction [19].

In this work, the oil has been obtained from almonds using two pressure systems: a screw press (SP) and a hydraulic press (HP). The performance of both methods, as well as the quality and composition characteristics of the almond oils obtained, both from the physical-chemical and organoleptic point of view, have been analyzed to determine the possible changes that occurred in the oil due to the extraction system. In addition, the nutritional characteristics of the defatted almond flour are characterized.

## 2. Materials and Method

### 2.1. Raw Material

Medium-sized almond kernels from the Comuna variety were purchased from local suppliers. The almonds were subjected to an exhaustive hygienic quality control to discard all those kernels with visible black spots or that were damaged in any way.

### 2.2. Oil Extraction and Elaboration of Defatted Flours

The oil extraction was done by pressure systems, using two type of presses: screw (Komet Oil Press CA59G—IBG Monforts Oekotec GmbH & Co. KG, Mönchengladbach, Germany) and hydraulic (MECAMAQ Model DEVF 80, Vila-Sana, Lleida, Spain).

To evaluate the influence of the temperature when the oil is extracted with the SP, five extraction temperatures were considered (50 °C, 75 °C, 100 °C, 150 °C, and 200 °C). This press also allows us to introduce variations in the rotation speed of the screw, which can accelerate the input of raw material that partly cool the system, reducing the effect of temperature in the oil extracted; so three different extraction speeds were tested (17 rpm, 49 rpm and 96 rpm).

On the other hand, the extraction by HP is carried out under room temperature conditions, so there is no influence of temperature. However, to evaluate the performance of this system related to oil yield and the possible differences in the obtained oil because of the increasing pressure, different extraction conditions were tested. Thus, two variables that may influence the extraction process were analyzed: pressure time (2 min, 3 min and 4 min) and extraction pressure (80 kg cm^−2^, 120 kg cm^−2^, and 160 kg cm^−2^).

After extraction, the oil was centrifuged to remove solid residues from the samples. The resulting oils were stored in dark glass bottles under refrigeration conditions to avoid oxidation until analysis.

In all cases, three repetitions of each group of conditions tested were done.

The defatted flours were obtained by the grinding of the pressing cake with a knife mill (Retsch GrindoMix 200, RETSCH GmbH, Haan, Germany) and the subsequent sieving through a sieve with a mesh diameter of 1 mm.

### 2.3. Analytical Determinations

Acidity, as % oleic acid, was determined by titration of an oil solution dissolved in ethanol/ether (1:1) with a 0.1 M ethanolic solution of potassium hydroxide [20].

The peroxide value, expressed in milli-equivalents of active oxygen per kilogram of oil (meq/kg), was determined as follows. A mixture of oil and chloroform-acetic acid was left to react with a solution of potassium iodide in darkness; the free iodine was then titrated with a sodium thiosulfate solution [20].

The coefficients K270 and K232 were calculated by absorbance at 270 and 232 nm, respectively, in a Jasco V-530 UV/VIS spectrophotometer (Jasco Analitica Spain, Madrid, Spain), and a path length of 1 cm. For K270, a 1% solution of oil in cyclohexane was used, while for K232 a 0.5% solution was used [20].

Total phenols were isolated by extracting a solution of oil in hexane three times with a water/methanol mixture (60:40). Folin–Ciocalteau reagents and sodium molybdate, 5% in 50% ethanol (Merck), were added in the appropriate proportions and the absorption of the solution was measured at 725 nm. The results have been expressed in mg of caffeic acid equivalents per kg of oil [21,22].

Oxidative stability was evaluated by the rancidity method [23] and was expressed as the oxidation induction time (hours), measured with the Rancimat 743 apparatus (Metrohm Co., Basel, Switzerland). A 3.5 g oil sample was used, and heated to 100 °C under an air flow of 10 L/h.

To determine the fatty acid composition (%), the methyl esters were prepared by vigorously shaking a solution of oil in hexane (0.2 g in 3 mL) with 0.4 mL of a 2 N methanolic potassium hydroxide solution, and analyzed by GC with a Hewlett-Packard (HP 6890) chromatograph equipped with a FID detector. A fused silica column (50 m long × 0.25 mm i.d.) was used, coated with an SGL-1000 phase (0.25 mm thickness; Sugerlabor). Helium was employed as a carrier gas with a flow through the column of 1 mL/min. The temperature of the injector and the detector were adjusted to 250 °C with an oven temperature of 210 °C. An injection volume of 1 mL was used (EEC Regulation 2568/91, in correspondence with the American Oil Chemistry Society (AOCS) Ch 2–91 method.

Sterols were determined with a Hewlett–Packard (HP 6890) gas chromatograph with a capillary column (25 m length × 0.25 mm i.d.) coated with SGL-5 (0.25 μm thickness; Sugerlabor). Working conditions were as follows: carrier gas, helium; flow through the column, 1.2 mL min^−1^; injector temperature, 280 °C; detector temperature, 290 °C; oven temperature, 260 °C; injection volume 1 μL (Regulation EEC 2568/91, corresponding to AOCS method Ch 6-91). Apparent β-sitosterol was calculated as the sum of β-sitosterol, Δ5,23-stigmastadienol, cholerosterol, sitostanol, and Δ5,24-stigmastadienol.

### 2.4. Proximate Analysis of Defatted Flours

The moisture content was calculated by measuring the weight loss after drying at 105 °C for at least 72 h, until constant weighing [24]. The protein content was calculated by multiplying the total nitrogen content, obtained by the Kjeldahl method [25], by a conversion factor of 4.38 [26]. To determine the ash content, the flours were calcined at 540 °C for at least 6 h, until constant weight loss. The crude fat content (ether extract) was determined by extraction with petroleum ether using the filter bag technique with the help of an Ankom XT10 extraction system [27]. For the determination of the crude fibre content, the Weende technique adapted to the filter bag technique was applied, subjecting the sample to two successive digestions with solutions of sulfuric acid and sodium hydroxide, using for this an Ankom 220 Fiber Analyzer equipment [28]. The total carbohydrate content was calculated by subtracting from the total weight of the flours the sum of the contents of crude protein, crude fat, moisture and ash [29]. Available carbohydrates (nitrogen-free extractives) result from subtracting the crude fiber content from the total carbohydrate content [24]. The energy value was calculated from the relative contents of protein, fat, and carbohydrates using the modified Atwater factors [24].

### 2.5. Sensory Analysis

Regarding the sensory analysis of almond oils, a test to measure the degree of consumer liking was carried out through the use of verbal hedonic scales and consumer judges (Affective Consumer Test). Affective tests are those in which the judge expresses his subjective reaction to the product, indicating, in the case of the degree of satisfaction measurement test, how much they like or dislike a food [30,31]. The panel consisted of at least 80 consumer judges. A nine-point verbal hedonic scale was used, ranging from −4 least preference to +4 highest preferences to assess color, odor, and taste. Each parameter was analyzed separately and the mean scores for the three sensory properties were calculated.

A preference test (affective test) was also carried out, in which the consumer judge must choose between two samples, being able to indicate, in the comments section, the reason for such choice. The preference test has been used to evaluate the preference of consumers regarding the type of press used (screw and hydraulic), once the best extraction conditions have been selected in each of the types.

Finally, a descriptive test has been used to describe the characteristics and the positive and negative attributes of the almond oil through a test with non-structural scales. These unstructured scales only have extreme points (minimum and maximum), and the judge must express his appreciation of the intensity of the attribute of a sample of food by marking a line between both extremes. The parameters analyzed were: color, fresh/roasted almond smell, fresh roasted almond flavor and sweetness, and presence of defects, such as burnt and rancid.

### 2.6. Statistical Analysis

Determinations in this study are means of triplicate measurements from three independent samples. Regarding the physical-chemical parameters, chemical composition and sensory attributes, statistical differences were estimated from the analysis of variance (ANOVA) test at the 5% level of significance and Duncan test (*p* < 0.05). Principal component analysis (PCA) was developed for the almond oil samples. All statistical analyses were carried out using the SPSS program.

## 3. Results and Discussion

### 3.1. Yield

**Figure 1:**
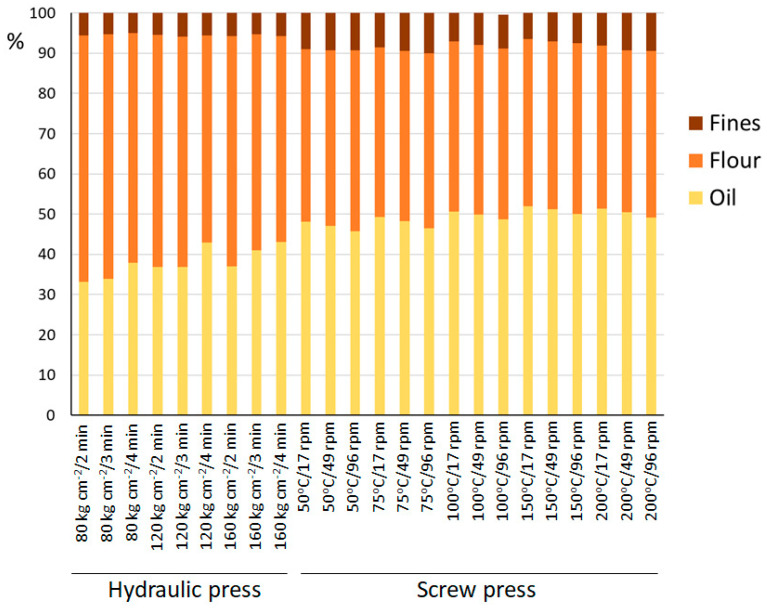
Yield in oil and flour, as well as the number of fines removed from the oil of the extracted almonds by SP, varying the temperature (50 °C, 75 °C, 100 °C, 150 °C, and 200 °C) and the extraction speed (17 rpm, 49 rpm, and 96 rpm) and HP, varying the pressure (80 kg cm^−2^, 120 kg cm^−2^, and 160 kg cm^−2^) and the extraction time (2 min, 3 min, and 4 min).

Figure 1 shows the yields obtained in oil, the resulting flour obtained by grinding the pressing cake, and the amount of residue removed from the oil (fines), which remain after oil centrifugation, and that are discarded. Both extraction methods were tested under different conditions: in the hydraulic press different combinations were made according to pressure applied and time, and in the screw press combinations of extraction temperature and rotation speed were tested. The oil obtained in the screw press is significantly higher (49.18% on average) than that obtained with the hydraulic press (37.94%). It can be explained by two main causes: the effect of temperature, which even when the press works at lower temperatures may reach high values [32], and, on the other hand, the greater interaction of the system with the almonds that are being pressed, because the rotational movement of the screw originates shear forces due to the friction that may contribute to the breaking of the structures of the parenchyma and the liposomes that contain the oil. Despite the high yield obtained when the screw press is used, there is still some oil remaining in the pressing cake, since the extractive efficiency that is achieved with pressure systems does not reach in any case that obtained when solvents are used. However, these methods only consist of mechanical extraction with no chemical reagents involved, so the oils obtained can be considered as virgin oils, which can be consumed directly without refining processes.

When a deeper view is paid to the samples extracted with the screw press, the rotation speed seems to be another determining factor that affects the oil extraction yield. A lower rotation speed is related to a longer time that the almonds are subjected to pressure, leading to a greater oil yield. The effect of the temperature of the heating ring in the oil yield is also remarkable, although when the temperature exceeded 100 °C, no significant differences were observed. The highest oil extraction yield (52%) was obtained when 150 °C and a rotation speed of 17 rpm were used in the screw press.

On the other hand, lower oil extraction yields were obtained when hydraulic press was used. In this case, a proportional increase in the oil extraction yield was obtained when higher pressure was applied or longer times for extraction was used. The highest oil yield with the extraction conditions tested in the hydraulic press was obtained when a pressure of 160 kg/cm^2^ was applied for 4 min (43.12%).

Consequently, the greater the oil extraction yield, the lower the amount of flour produced. Thus, the average content of flour obtained when the hydraulic press was used was 56.39 g/100 g, while for the screw press, an average of 42.27 g/100 g were obtained.

From an industrial point of view, the highest oil yield is obtained with the SP when it operates at temperatures of 100–150 °C, which has the additional advantage of operating continuously, due to the lower costs it entails.

### 3.2. Physico-Chemical Analysis of Almond Oil

**Figure 2:**
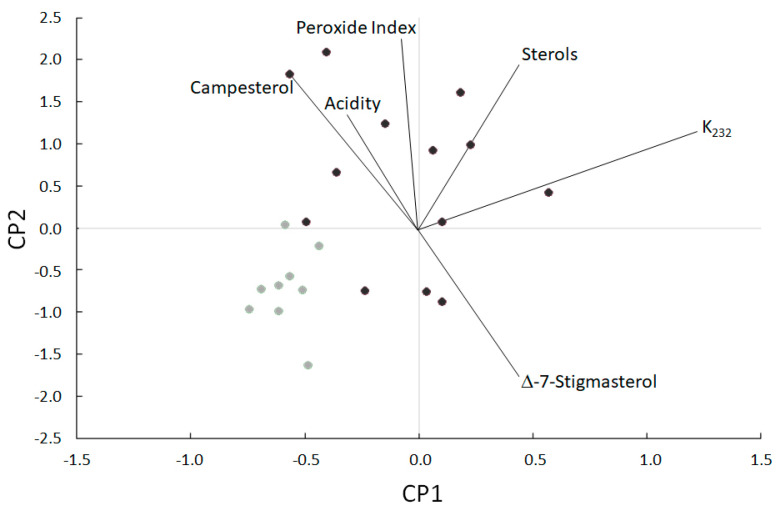
Principal component analysis with the physicochemical parameters, stability and parameters for regulated quality in almond oils obtained under different pressing systems: hydraulic press (light grey circles) and screw press (dark grey circles).

Figure 2 shows the principal component analysis applied to almond oil produced with both pressure systems. For this analysis, the chemical composition data and the physicochemical quality and stability parameters have been used. Functions 1 and 2 describe 74.85% of the variance. In this analysis, the effect of temperature during extraction is clearly appreciated. The samples extracted with the screw press clearly separate from those extracted with the hydraulic press, mainly due to an increase in certain quality parameters such as the K232 value, acidity, or the peroxide value.

The samples obtained by HP appear more compact, without significant differences, while those obtained with SP present greater diversity with some significant differences, which are discussed below.

The main parameters used to evaluate the quality of oil are the acidity (free fatty acids), the extinction coefficients K232 and K270 measured by the UV absorbance at 272 and 270 nm, and the peroxide value. The acidity values are very low in all the conditions tested independently of the type of press (0.10–0.20%), which indicates the freshness and high quality of the oils obtained by physical methods. Although high temperatures can be used in the screw press to improve extraction, it is a method that is considered cold extraction, since the heat treatment to which the raw material is subjected is not aggressive for the oil. Acidity values were below 0.2%, even when screw press with the highest temperature tested was used, which indicates that the extraction process did not greatly affect the quality of the oil obtained. Compared with other most-used oils, for example, in olive oil, an acidity value below 0.8% is considered for the highest quality oil (extra virgin olive oil). These values are in line with those previously reported [10,32,33].

**Figure 3:**
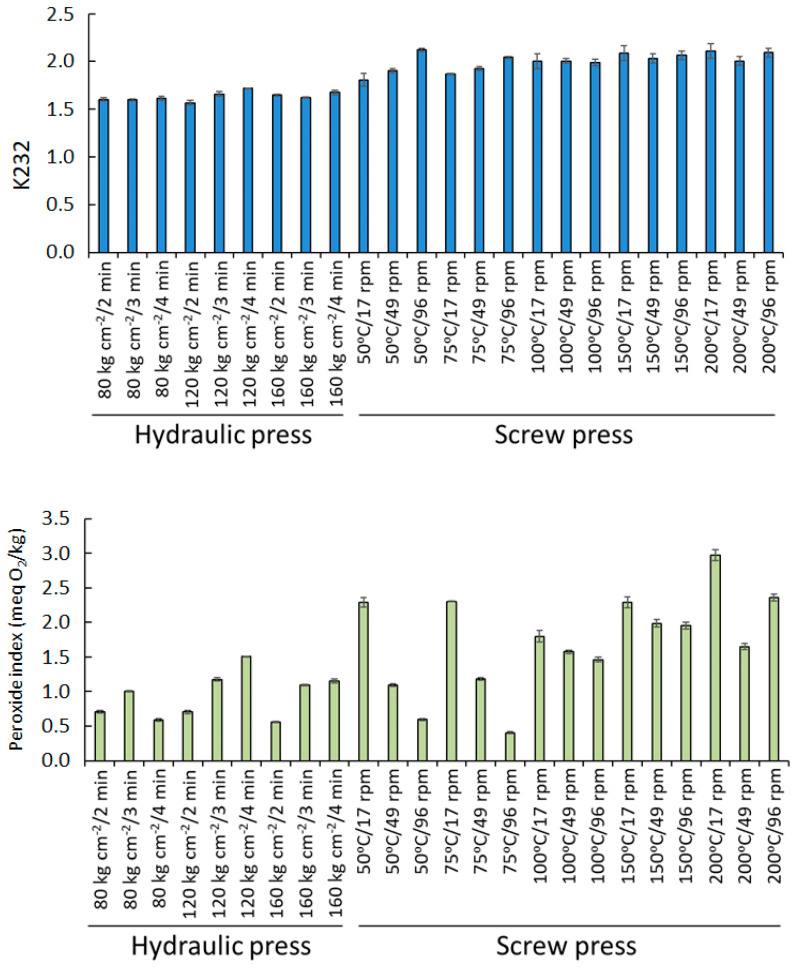
Parameters of regulated physicochemical quality (K232 and peroxide value) in almond oils obtained under different pressing systems and conditions.

The K232 values are significantly higher in the samples obtained with the screw press (2.11 on average) compared to those obtained by means of the hydraulic press (1.65) (Figure 3), which would be explained by the higher temperature and longer exposure time. However, there are no significant differences between the samples obtained at different temperatures in the screw press, probably because the temperature differences inside the screw are much lower than the nominal ones when working continuously [32]. Regarding the extinction coefficient, K270 is the only qualitative index where no differences between the two systems were found, which may be explained by the fact that the K270 is indicative of more advanced oxidation states, which are not reached during the extraction procedure.

In the Figure 3, the peroxide values of the oil samples extracted with the two pressure systems under the conditions tested are shown. This value is indicative of the initial oxidation state of the oils, which is the result of the presence of hydroperoxides resulting from the oxidation of fatty acids. This process is greatly affected by temperature that increases the speed of oxidation reactions, which can be observed in the oil samples extracted. Peroxide value increases when higher temperatures are used for the extraction, but also when rotation speed is low, due to a higher time that almonds are subjected to the effect of temperature. When the temperatures used for the oil extraction with the screw press are lower than 100 °C and the rotation speed is high, the peroxide value of the oils is similar to that obtained with the hydraulic press where extraction is done under room temperature. However, when the temperature of the screw press is higher than 100 °C the peroxide value of the oils increases independently of the rotation speed used.

Regarding chemical composition, the results obtained show a fatty acid profile for almond oil (Table 1) characterized by its low content of saturated versus monounsaturated fatty acids with a predominance of oleic acid (68.6–71.3%), followed by linoleic (19.4–21.4%) and palmitic (6.3–6.7%). Just these three acids represent approximately 97% of the fatty acids, in agreement with other previous reports [34,35,36]. Traces of lauric, myristic, linolenic, behenic, and lignoceric acids have also been found (<0.1%) (not shown in the table). No significant differences on the fatty acid profile were found between the two extraction systems, or within the different conditions tested for each extraction system. The low content in saturated fatty acids of these oils is noteworthy (<10%), which is even lower than in other appreciated and widely oils such as extra virgin olive oil.

**Figure 4:**
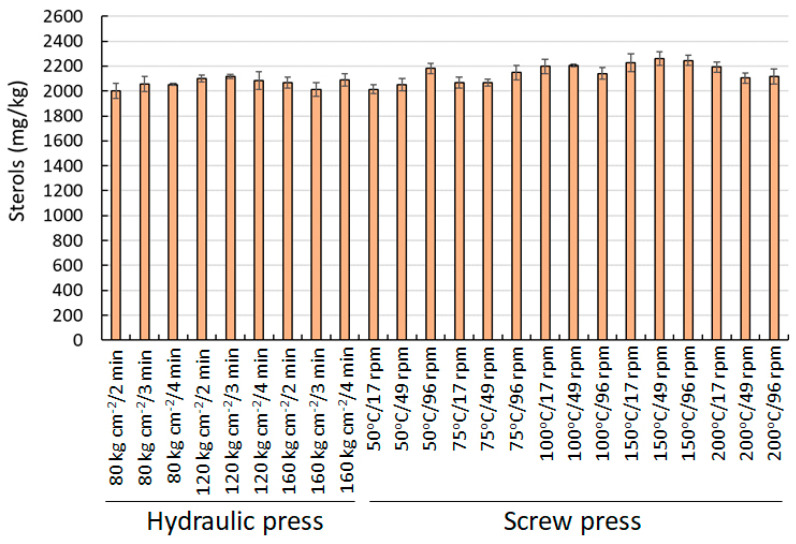
Sterol content of almond oils obtained by SP and HP (mg/kg).

Figure 4 shows the average value in the content of total sterols for the samples extracted with the hydraulic and the screw press. These compounds are particularly important for their high nutritional value and health benefits. Phytosterols can reduce blood cholesterol, risk of certain types of cancer and enhance immune function [37]. The low value of cholesterol (0.1 mg/100 g) stands out, together with the presence of other phytosterols, mainly β sitosterol (95.40–95.60%), campesterol (2.60–2.80%) and stigmasterol (0.60%), with values similar to those found by [32,38,39,40,41]. Between the two extraction systems, only significant differences were found in the number of total sterols, which were higher in the case of the screw press with an average of 2153 ± 84 mg/kg, compared to 2069 ± 55 mg/kg in the hydraulic press. Within the operational conditions tested for each system, no significant differences were observed in HP in any of the compounds analyzed. As regards extraction with SP, a higher amount of total sterols was observed when high temperature was used (150 °C), although its composition remained unchanged.

Phenolic compounds also have important biological activities due to their high antioxidant capacity, for which healthy effects have been described [6]. Several in vitro assays developed to assess total antioxidant capacity in foods have focused on the effect of polyphenols. Data from large observational studies show that regular nut consumption is associated with a reduced risk of several conditions in which oxidative stress may play a role, including coronary heart disease, hypertension, type 2 diabetes, inflammation, and endothelial dysfunction [42].

Thus, the presence of phenolics gives these oils positive nutritional characteristics, which make them interesting candidates to be included in a healthy diet. Regarding extraction, no significant differences have been found for the total phenolic compounds content, neither for both systems nor for the different extraction conditions evaluated for each system. The average value for the total polyphenol content for HP system was 19.79 mg of caffeic acid equivalents per kg of oil; meanwhile, for SP, the system was 20.40 mg of caffeic acid equivalents per kg of oil.

The oxidative stability measures the susceptibility of the oil to lipid oxidation, providing some indication of its shelf life. Due to its antioxidant capacity, phenolic compounds may play an important role on the oxidative stability of oils. In the case of almond oils extracted by pressure, the average values of oxidative stability obtained ranged between 19.70 ± 1.10 h (SP) and 20.09 ± 0.64 h (HP). In many other edible oils, heat treatments before or during extraction may increase oxidative stability values, because temperature may break certain bonds that facilitate the extraction of antioxidant compounds. However, in the case of almond oils, this fact does not seem to have this effect, as there is no significant difference when data about oxidative stability from both extraction systems are compared.

Regarding the quality and chemical composition, the oils obtained by HP showed better quality indices, as they are subjected to a less aggressive treatment, but lower content in total sterols. Fatty acid pattern was not affected by the pressing system. The fat-soluble bioactive compounds present make it an oil of great biological and nutritional importance.

### 3.3. Sensory Evaluation

**Figure 5:**
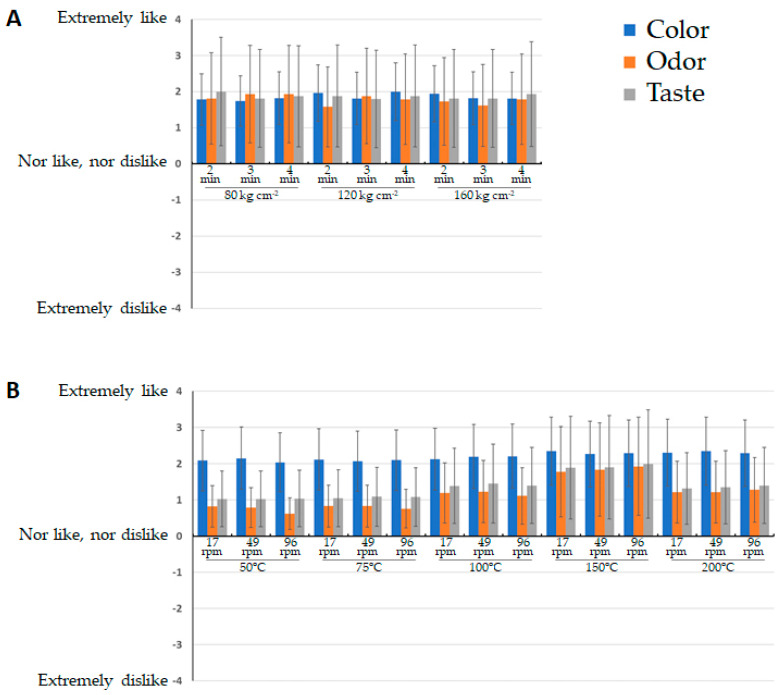
Sensory parameters evaluated (color, odor, and taste) in the test to measure the degree of satisfaction through the use of verbal hedonic scales of the oil samples. (**A**) Oils obtained by hydraulic press. (**B**) Oils obtained by screw press.

All the almond oil samples extracted with the two pressure systems under different extraction conditions were subjected to sensory evaluation to evaluate the influence of the operational conditions in the organoleptic characteristics of the resulting oils. The results of the sensory tests to measure the degree of satisfaction are shown in Figure 5, where the values correspond to the average scores for color, odor, and taste from 80 consumer judges.

Consumers positively valued all the almond oils extracted using both pressure systems, as the average values for the three parameters evaluated were higher than 0 in all cases. Regarding the oils obtained using the hydraulic press, the extraction conditions did not affect the organoleptic characteristics of the oils, since no significant differences have been detected for none of the sensory parameters analyzed (Figure 5A). When the oils were extracted with the screw press, significant differences were found for the sensory attributes odor and taste. The most appreciated were those oils obtained at temperatures of 150 °C, since this temperature provides oils with an odor that reminds roasting almonds and a more intense taste, possibly because this is the most common way of consuming almonds (snacks). However, the oils obtained at lower temperatures (50 °C and 75 °C) were the least valued, possibly due to the malfunction of the screw press at low temperatures, that may originate the appearance of less pleasant tastes and odors, which are not appreciated by consumers (Figure 5B). When temperatures are higher, the oil becomes less viscous, flows better, and results in better operation of the entire system.

To evaluate the consumers’ preference for the oils extracted by the two pressure systems, a preference test was carried out, in which the consumer judge must choose between one of the two oils. In this test, the oils used were those that obtained the best scores within each extraction system in the previous sensory test. The results obtained showed that no significant differences were found to any sensory attribute between the samples extracted with different types of press (screw and hydraulic). Sensory characteristics of the oils obtained are different, since the oils obtained with the screw press are subjected to a thermal treatment during extraction, which gives them a greater intensity in both parameters the odor and the taste. However, the scores of both oils were similar, since in one case a more intense smell and flavor with some touches of roasted almonds were appreciated in the case of the oil extracted with the screw press, while in the other hand, the natural odor and taste of the oil obtained by the hydraulic press was appreciated. Thus, it is advisable to market both types of oils, since they are differentiated products that can reach different types of consumers according to the different sensory characteristics.

Finally, the almonds oils were subjected to a descriptive characterization, made by trained judges. The almond oils extracted using the screw press has a golden yellow color, with the smell of roasted almonds and a sweet taste with touches of roasted almonds, when the extraction temperature was high (from 100 °C). When the extraction temperature was lower than 100 °C, some defects such as plastic odor appeared due to a malfunction of the press. On the other hand, the oils extracted using the hydraulic press showed a light yellow color with greenish tones, and a smell and flavor that reminds of fresh almonds, combined with clear sweet touches.

The operational conditions tested in both presses did not greatly affect the chemical composition of the oils obtained, as fatty acid pattern was similar independently of the different conditions tested or the press used. Only minor compounds such as sterols showed small differences.

When oils were subjected to sensory analysis based on consumer preferences by means of a preference test, where judges should show their preference for one of the oils (HP or SP), both obtained similar valuations, as 51% of consumers preferred the oil extracted by SP, while 49% of the consumers showed their preference for the oil from HP. The consumers who choose the oil obtained by HP valued its natural aroma, while the ones who choose the oil from SP valued the higher intensity in odor and its taste to roasted almonds. Thus, although the raw matter is the same, the oil extraction using different pressing systems results in clearly differentiated products regarding sensory properties.

### 3.4. Proximate Composition of Almond Defatted Flour

At present, there is great interest in the use of prebiotics as ingredients of functional foods to manipulate the composition of microbiota colonies and improve health [43]. For this reason, the chemical characterization of the defatted almond flour obtained by grinding of the pressing cake has been carried out. Table 2 shows the average values of the flour composition from both extraction systems. The great differences in oil yield found between the two systems are transferred to the composition of the flours leading to a high fat content in the flour obtained from the hydraulic press (27.40%). This causes the proportions to vary in the rest of the components, which present significant differences in all of them. Karaman et al. [44] have also analyzed flours after cold pressing and separation of different edible oils, among which was almond. The oil, protein, and crude fiber content of the almond flour was 8.8%, 49%, and 5.9%, respectively, so it could be used to enrich the protein content of various food products. Defatted almond flours are especially rich in protein content (44.46% in hydraulic press; 55.88% in screw press). They also had a high proportion of total carbohydrates, among which fiber showed values over 3%. Considering nutritional composition, the almond defatted flours are an energy rich ingredient, with higher energy values in the flour obtained after hydraulic extraction due to the high amount of fat that remains in the pressing cake.

Defatted almond flour obtained from the press cake that results from the oil extraction process has a high nutritional value, especially due to its high protein content. In addition, it is rich in fiber, and the content of carbohydrates and the resulting oil make this flour an energy rich ingredient.

Moreover, some other minor compounds with antioxidant activity of the flours and their extracts promote their used as functional ingredients, which may be added to improve human diet, or as a natural antioxidant for products containing oils and fats [45].

Other alternative uses of defatted almond flours have also been explored. The use of these flours as a novel nutritional supplementation for cultivated edible mushrooms has been tested with positive results [17].

## 4. Conclusions

Both pressure extraction systems (hydraulic and screw) are a good technology to obtain high quality almond oils, with a low content of free fatty acids and peroxides, leading to the production of a virgin oil that can be consumed directly, without refining. In addition, these extraction systems allow the presence of fat-soluble bioactive compounds in the oil, which has been described to have important antioxidant properties, and that increase their nutritional value.

The main difference between both extraction systems is obtained in the oil extraction yield, that is significantly higher in the screw press operating at extraction temperatures of 100–150 °C. It has the additional advantage of operating continuously, due to the lower costs it entails.

The oils obtained by HP showed better quality indices (acidity, peroxide value, K232, K270), as they are obtained at room temperature, and no effect of higher temperatures are observed in these oils. Regarding chemical composition, no differences were observed in the fatty acid profile of the oils, characterized by the predominance of unsaturated fatty acids (>90%). The oils obtained by screw press showed a slight increase in total sterols.

Although chemical composition of the oils is quite similar, the results obtained from the sensory analysis showed two differentiated products. The oils obtained from the screw press showed an odor and taste of roasted almonds, while the oils from the hydraulic press were characterized by a lighter odor and taste of fresh almonds. Both oils were equally appreciated by consumers, so both products could be marketed, targeting different consumer sectors.

The grinding of the pressing cake originates a partially defatted almond flour, which may be considered as a by-product of the extraction process. These defatted flours showed a high content in protein and fiber, and with a higher content in fat in the flour obtained from the hydraulic press. The nutritional characteristics of these by-products make them promising ingredients to be included in the formulation of novel functional foods.

## Figures and Tables

**Table 1:** Fatty acid composition. Average value of samples from different extraction systems (%).

	Hydraulic Press	Screw Press
Palmitic acid C16:0	6.4 ± 0.21	6.4 ± 0.41
Palmitoleic acid C18:0	0.5 ± 0.01	0.6 ± 0.08
Margaric acid C17:0	0.1	0.1
Margaroleic acid C17:1	0.1	0.1
Stearic acid C18:0	2.1 ± 0.06	2.1 ± 0.05
Oleic acid C18:1	71.1 ± 1.16	70.6 ± 1.52
Linoleic acid C18:2	19.5 ± 0.55	19.8 ± 0.91
Linolenic acid C18:3	0.1	<0.1
Arachidic acid C20:0	0.1	0.1
Gadoleic acid C20:1	0.1	0.1
Saturated Fatty acids	9.2 ± 0.28	9.3 ± 0.54
Unsaturated Fatty acid	90.9 ± 1.71	90.6 ± 2.43

**Table 2:** Proximate composition of defatted almond flour obtained by the grinding of the pressing cake from the hydraulic and screw presses. only those conditions that were best rated by consumers for each pressing system are shown to avoid unnecessary data repetition.

	Hydraulic Press	Screw Press
Humidity (%)	8.36	9.70
Nitrogen (%)	7.11	8.94
Protein (%)	44.46	55.88
Ash (%)	5.93	7.39
Crude fiber (%)	3.13	3.86
Crude fat (%)	27.40	8.63
Total carbohydrates (%)	22.21	28.10
Available carbohydrates (%)	19.09	24.24
Energy value (kcal/100 g)	474.00	380.50
